# Diabetes impairs an interleukin-1β-dependent pathway that enhances neurite outgrowth through JAK/STAT3 modulation of mitochondrial bioenergetics in adult sensory neurons

**DOI:** 10.1186/1756-6606-6-45

**Published:** 2013-10-24

**Authors:** Ali Saleh, Subir K Roy Chowdhury, Darrell R Smith, Savitha Balakrishnan, Lori Tessler, Emily Schartner, Andre Bilodeau, Randy Van Der Ploeg, Paul Fernyhough

**Affiliations:** 1 Division of Neurodegenerative Disorders, St. Boniface Hospital Research Centre, R4048 - 351 Tache Ave, Winnipeg, MB R2H 2A6, Canada; 2 Department of Pharmacology & Therapeutics, University of Manitoba, Winnipeg, MB R3E 0T6, Canada

**Keywords:** Neurotrophic factor, Axon regeneration, Bioenergetics, Neuropathy, Dorsal root ganglia

## Abstract

**Background:**

A luminex-based screen of cytokine expression in dorsal root ganglia (DRG) and nerve of type 1 diabetic rodents revealed interleukin-1 (IL-1α) and IL-1β to be significantly depressed. We, therefore, tested the hypothesis that impaired IL-1α and IL-1β expression in DRG may contribute to aberrant axon regeneration and plasticity seen in diabetic sensory neuropathy. In addition, we determined if these cytokines could optimize mitochondrial bioenergetics since mitochondrial dysfunction is a key etiological factor in diabetic neuropathy.

**Results:**

Cytokines IL-1α and IL-1β were reduced 2-fold (p<0.05) in DRG and/or nerve of 2 and 5 month streptozotocin (STZ)-diabetic rats. IL-2 and IL-10 were unchanged. IL-1α and IL-1β induced similar 2 to 3-fold increases in neurite outgrowth in cultures derived from control or diabetic rats (p<0.05). STAT3 phosphorylation on Tyr705 or Ser727 was depressed in DRG from STZ-diabetic mice and treatment of cultures derived from STZ-diabetic rats with IL-1β for 30 min raised phosphorylation of STAT3 on Tyr705 and Ser727 by 1.5 to 2-fold (p<0.05). shRNA-based or AG490 inhibition of STAT3 activity or shRNA blockade of endogenous IL-1β expression completely blocked neurite outgrowth. Cultured neurons derived from STZ-diabetic mice were treated for 24 hr with IL-1β and maximal oxygen consumption rate and spare respiratory capacity, both key measures of bioenergetic fidelity that were depressed in diabetic compared with control neurons, were enhanced 2-fold. This effect was blocked by AG490.

**Conclusions:**

Endogenous synthesis of IL-1β is diminished in nerve tissue in type 1 diabetes and we propose this defect triggers reduced STAT3 signaling and mitochondrial function leading to sup-optimal axonal regeneration and plasticity.

## Introduction

Peripheral nerve injury is associated with an inflammatory response at the site of damage that is associated with enhanced expression of cytokines such as interleukin-1β (IL-1β), TNF-α and IL-6 [1-3]. Nerve damage, and associated Wallerian degeneration, causes the secretion of IL-1β by Schwann cells and invading macrophages leading to a cascade of events, in part leading to increased expression of nerve growth factor (NGF) by fibroblasts [4-7]. The induction of NGF provides neurotrophic support for regenerating nerve fibers but this inflammatory process also triggers the development of hyperalgesia [8,9].

Recent studies reveal that IL-1β may directly target sensory neurons. Adult sensory neurons express receptors for cytokines, such as LIF [10], IL-6 [11] and IL-1β [12] and these cytokines undergo enhanced expression within the dorsal root ganglia (DRG) upon nerve damage [13,14]. IL-1β is synthesized by DRG sensory neurons [12] and regulates axonal regeneration following peripheral nerve injury *in vivo*[7,15] and *in vitro*[6,16].

The JAK/STAT3 pathway is activated after nerve injury in DRG and motor neurons and maintains neuronal survival and drives axon regeneration [17-23]. Phosphorylation on Tyr705 and transcriptional activation of STAT3 is modulated by cytokines, including IL-1β [2,24,25] and growth factors [26,27]. STAT3 phosphorylation on Ser727 regulates its translocation to the mitochondria and modulates the activity of electron transport Complex I [28-30]. In PC12 cells the interaction of STAT3 phosphorylated on Ser727 with mitochondria was associated with NGF induction of neurite outgrowth [31].

High ATP consumption by growth cones during motility instills a need for optimal mitochondrial function to maintain axon regeneration and plasticity [32,33]. Mitochondrial dysfunction has been proposed as a central mediator of development of many neurodegenerative diseases [34,35], including neurological and other diseases associated with diabetes [33,36,37]. Recently, we have demonstrated that sensory neurons of diabetic rodents exhibit an abnormal mitochondrial phenotype that contributes to the etiology of diabetic neuropathy [38,39]. Recent studies reveal reduced cytokine expression in DRG and nerve of diabetic rodents [40] and the ability of neuropoietic cytokines to enhance mitochondrial bioenergetics [33]. We, therefore, tested the hypothesis that impaired IL-1β expression in DRG may contribute to aberrant axon regeneration and plasticity seen in diabetic sensory neuropathy.

## Results

### IL-1β protein levels are diminished in DRG and nerve tissue under a diabetic state

DRG and nerve tissue from 2 or 5 month STZ-induced diabetic rat cohorts were collected and analyzed for a panel of cytokines. Table 1 provides data on levels of IL-1α, IL-1β, IL-10 and IL-2 in DRG, sciatic nerve and tibial nerve. IL-1α and IL-1β protein levels were significantly reduced in lumbar DRG and in sciatic nerve at 2 and 5 months of diabetes (*p<0.05 vs diabetic). IL-1β protein levels were also significantly reduced in tibial nerve, however, IL-1α levels were unchanged in tibial nerve at 2 months. IL-2 and IL-10 were unchanged in any tissue at 2 and 5 months. At 2 and 5 months, the diabetic rats were hyperglycemic, exhibited loss of weight and were hypoalgesic (enhanced thermal withdrawal latency) compared with age matched control animals (presented previously in [40]).

**Table 1:** Effect of 2 or 5 month STZ-diabetes on cytokine levels in rat DRG and sciatic nerve

	**IL-1α**	**IL-1β**	**IL-10**	**IL-2**
**DRG**	**2 Months**			
Control	0.97 ± 0.1*	60.35 ± 15.4*	17.53 ± 6.0	4.53 ± 1.38
Diabetes	0.36 ± 0.05	28.38 ± 2.1	12.65 ± 3.6	2.63 ± 0.57
**Sciatic nerve**				
Control	22.64 ± 7.3*	63.93 ± 18.2*	9.78 ± 3.5	3.09 ± 0.28
Diabetes	9.55 ± 3.7	34.05 ± 6.3	13.87 ± 7.1	4.98 ± 0.90
**Tibial nerve**				
Control	25.0 ±1.7	102.4 ± 17.7*	31.79 ± 17.7	4.64 ± 3.04
Diabetes	19.46 ±9.5	45.41 ± 8.5	39.64 ± 3.9	6.12 ±2.53
**DRG**	**5 Months**			
Control	1.99 ± 0.6*	75.7 ± 10.9*	ND	5.99 ± 2.93
Diabetes	0.68 ± 0.1	32.1 ± 5.2	ND	1.93 ± 0.63
**Sciatic nerve**				
Control	15.9 ± 2.5*	64.15 ± 8.1*	ND	2.08 ± 0.77
Diabetes	3.6 ± 1.1	25.68 ± 5.3	ND	2.61 ± 1.15
**Tibial nerve**				
Control	17.0 ± 2.46*	105.4 ± 14.8*	ND	3.81 ± 1.63
Diabetes	3.4 ± 1.02	30.74 ± 6.6	ND	3.25 ± 0.94

Cytokine protein levels were determined using a Luminex machine with the BioRad 9-plex cytokine kit and are expressed in pg/mg total protein. A standard curve was generated for each cytokine and all values were derived from the linear aspect of the curve. Values are mean ± SD, n=4-6. *P<0.05 vs diabetic (Students *t*-Test).

### IL-1β elevates neurite outgrowth in adult rat sensory neurons

**Figure 1:**
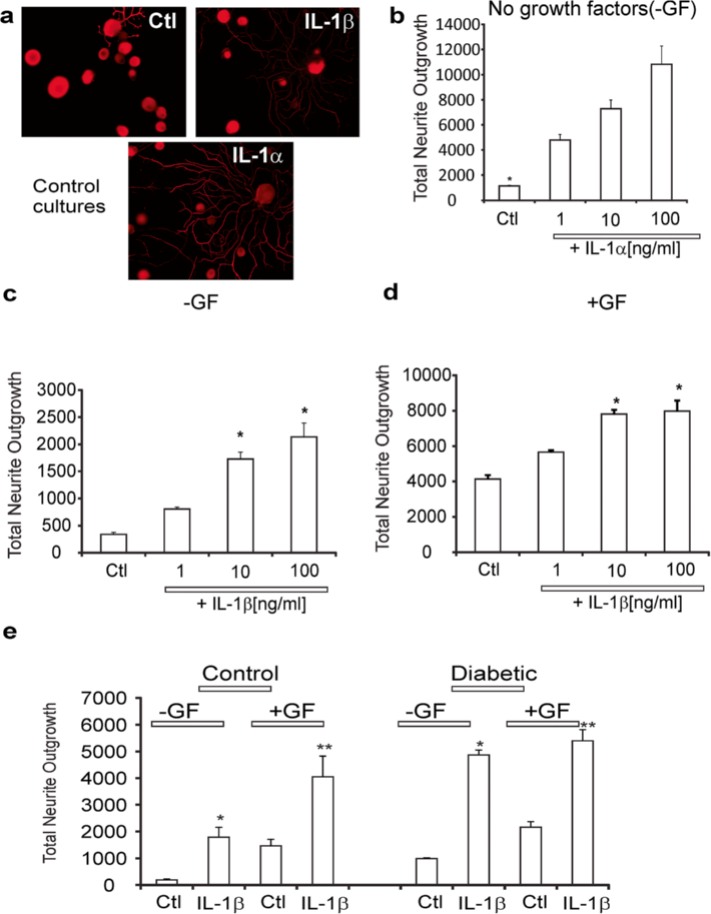
**IL-1α and IL-1β significantly increased neurite outgrowth in normal and diabetic neurons.** In **(a)** images of cultures stained for neuron-specific β-tubulin III are shown for untreated control (Ctl), treated for 24 h with IL-1β (10 ng/ml) or treated with IL-1α (10 ng/ml). Size marker indicates 100 μm. In **(b)** is shown total neurite outgrowth of neurons in response to a range of IL-1α concentrations grown for 24 h under defined conditions without neurotrophic growth factors (GF). Values are means ± SEM (n = 3 replicates); *p < 0.05 vs control (oneway ANOVA with Dunnett’s test). In **(c)** is shown total neurite outgrowth of neurons in response to a range of IL-1β concentrations without neurotrophic growth factors. Values are means ± SEM (n = 3 replicates); *p < 0.05 vs control (oneway ANOVA with Dunnett’s test). In **(d)** is shown total neurite outgrowth of neurons in response to a range of IL-1β concentrations in the presence of low dose cocktail of neurotrophic growth factors. Values are means ± SEM (n = 3 replicates); *p < 0.05 vs control (oneway ANOVA with Dunnett’s test). In **(e)** is shown total neurite outgrowth for sensory neurons isolated from control or 3–5 month STZ-diabetic rats cultured for 1 day ± IL-1β (at 10 ng/ml). Cultures were performed with or without neurotrophic factors. Values are the means ± SEM (n = 3 replicates). For control or diabetic *p < 0.05 vs control (Ctl) and **p < 0.05 vs Ctl (two-way ANOVA with Bonferroni’s test).

The ability of IL-1β to modulate adult sensory neuron phenotype was investigated. Adult rat DRG sensory neurons derived from normal rats were dissociated and grown under defined conditions and treated with a range of IL-1α and IL-1β concentrations for 24 h. Cultures were fixed, stained for neuron specific β-tubulin III (Figure 1a), and the levels of total neurite outgrowth were quantified. These experiments were performed in the presence or absence of a cocktail of neurotrophic factors. IL-1α and IL-1β stimulated neurite outgrowth in a dose-dependent manner under both conditions (Figure 1b, c, d) with a significant effect starting at 10 ng/ml for both cytokines (*p<0.05 vs control). There were three to five fold increases in neurite outgrowth when cultures were grown without neurotrophic growth factors (Figure 1b and c). In the presence of growth factors the IL-1β-dependent induction of neurite outgrowth was approximately 2-fold (Figure 1d). Cultures were then prepared from age matched control or 3–5 month STZ-induced diabetic rats and impact of IL-1β (10 ng/ml) on neurite outgrowth was analyzed. Figure 1e shows that IL-1β was able to elevate neurite outgrowth in DRG neurons from normal and diabetic rats (*p<0.05 vs control).

### IL-1 receptor type 1 and P-STAT3-Tyr705 are expressed in control rat DRG neurons

**Figure 2:**
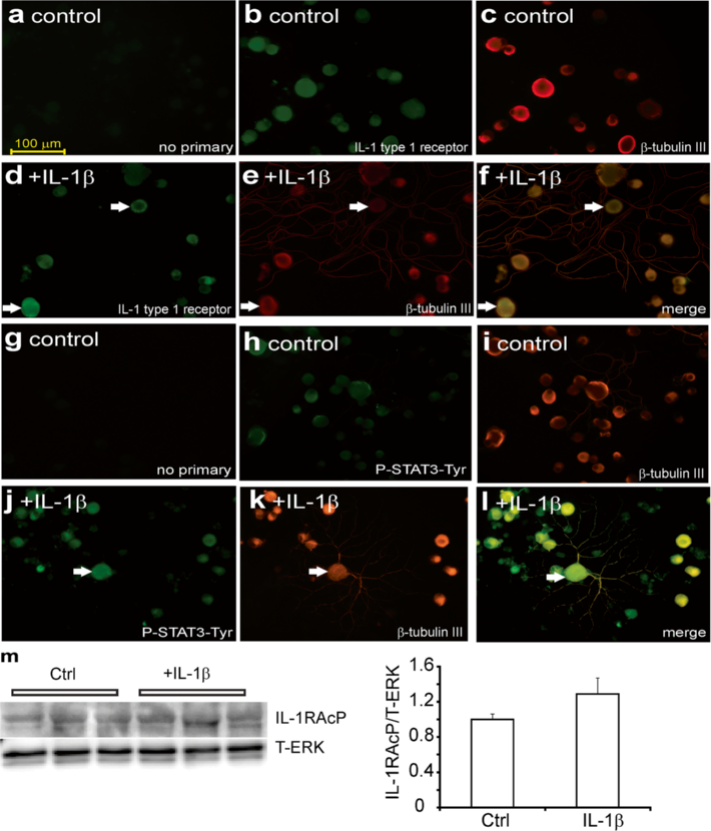
**Immunocytochemical staining for the IL-1 type 1 receptor and P-STAT3-Tyr705 in control neurons.** DRG sensory neurons from normal rats were cultured for 1 day in the presence of low dose of neurotrophic growth factors and were fixed and immunostained with **(a, g)** no primary antibody, or stained for **(b)** IL-1 type 1 receptor antibody or **(c)** β-tubulin III or **(h)** P-STAT3-Tyr705 or **(i)** β-tubulin III. Cultures were also treated for 24 h with IL-1β (10 ng/ml) and stained for **(d)** IL-1 type 1 receptor or **(e)** β-tubulin III or **(j)** P-STAT3-Tyr705 or **(k)** β-tubulin III. **(f)** and **(l)** are merge of **(d+e)** and **(j+k)** with secondary staining for neuron-specific β-tubulin III. The white arrows indicate co-localization of IL-1 type 1 receptor or P-STAT3-Tyr705 with neuron-specific staining. The bar = 100 μm. **(m)** Western blot for IL-1 type 1 receptor comparing control and treated with IL-1β cultures for 30 minutes in vitro with quantification. Expression levels are expressed relative to T-ERK and are means ± SEM (n = 3 replicates); no significant difference between Ctrl and +IL-1β.

We investigated the localization of IL-1 receptor type 1 and P-STAT3-Tyr705, as an indicator of cytokine signaling, in control rat DRG neurons. Adult rat DRG sensory neurons were cultured for 24 h with low dose of neurotrophic factors, in the presence or absence of IL-1β at 10 ng/ml. Cultures were fixed, stained for IL-1 receptor type 1 or P-STAT3-Tyr705 and neuron-specific β-tubulin III (Figure 2). Immunocytochemical staining for the IL-1 receptor type 1 revealed that all neuronal sizes expressed the receptor with no evidence of non-neuronal expression (Figure 2b, d and f). Expression of the IL-1 receptor type 1 by cultured neurons was not significantly altered by treatment with IL-1β although a small cohort of neurons expressed elevated IL-1 receptor type 1 in response to IL-1β (indicated by white arrows in Figure 2d-f). Background staining for P-STAT3-Tyr705 was very low in control cultures but was expressed in all neurons (Figure 2h and j). Treatment with IL-1β caused an increase in P-STAT3-Tyr705 staining (Figure 2j and k). In Figure 2m we used Western blotting to confirm that neurons expressed the IL-1 receptor type 1 and that expression was not significantly affected by IL-1β treatment.

### IL-1β activates the JAK/STAT pathway

**Figure 3:**
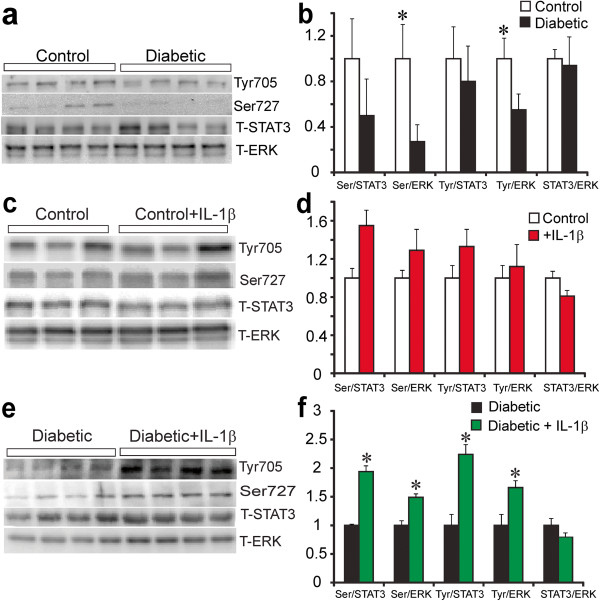
**STAT3 phosphorylation is impaired in DRG in diabetes and enhanced by IL-1β in neuron cultures.** In **(a)** is shown Western blot of DRG samples from control and 5 month STZ-diabetic mice. In **(b)** expression levels are indicated and are presented relative to T-ERK or T-STAT3 and are means ± SEM (n = 3–4 mice) *P < 0.05 vs control. In (**c** and **e**), are shown representative Western blots of DRG from normal or diabetic rats cultured for 1 day at low dose of neurotrophic growth factors and stimulated for 30 minutes with IL-1β at (10 ng/ml). In (**d** and **f**) are bar charts showing levels of phosphorylation of STAT3 on Ser-727 (Ser) or Tyr-705 (Tyr) presented relative to total ERK or total STAT3. *P < 0.05 vs untreated. Values are mean ± SEM, n = 4 (two-way ANOVA with Bonferroni’s test). Bar charts b, d, and e also show total STAT3 levels relative to T-ERK.

The ability of IL-1β to stimulate the JAK/STAT pathway in cultured adult sensory neurons was tested using quantitative Western blotting. DRG tissues from control and diabetic mice were homogenized and Western blotted for STAT3 phosphorylation on Tyr705 and Ser727. Levels of STAT3 phosphorylation on Tyr705 and Ser727 were significantly diminished in diabetic compared to control mice when normalized to T-ERK (the expression of T-ERK is never affected by the diabetic state in DRG; Figure 3a). The level of P-STAT3-Ser727 was reduced relative to T-STAT3, however, P-STAT3-Tyr705 was not altered (Figure 3b). Levels of T-STAT3 relative to T-ERK did not change in diabetes. Adult DRG sensory neurons derived from normal and diabetic rats were cultured for 24 h and treated with 10 ng/ml IL-1β for 30 min and neurons were lysed and levels of phosphorylated STAT3 on Tyr705 and Ser727 were determined (Figure 3c and e). IL-1β induced an approximate 1.5 to 2-fold increase in P-STAT3-Tyr705 or Ser727 phosphorylation at 30 min (Figure 3f) in diabetic cultures when presented relative to T-ERK or T-STAT3. We did not see any significant effect of IL-1β on STAT3 phosphorylation on either epitope in control cultures at 30 min, however, there was a trend towards a 25-40% increase (Figure 3d). Note in Figure 3e (and 4e later) that T-ERK signal revealed only one band. This was due to changes in lot number of antibody from the commercial source. In our hands, the 42 or 44 kDa ERK bands never exhibit any differential change in expression under a variety of conditions in vivo and in vitro.

### IL-1β modulates neurite outgrowth via JAK/STAT pathway

**Figure 4:**
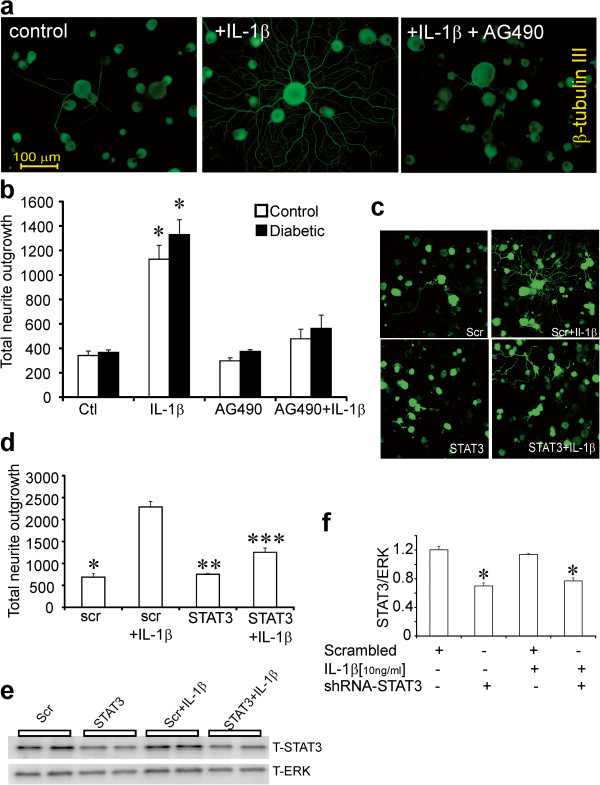
**Blockade of STAT3 signaling inhibits IL-1β-mediated neurite outgrowth.** DRG sensory neurons from normal and diabetic rats were cultured for 1 day in the presence of low dose neurotrophic growth factors (to improve viability in presence of AG490) and were exposed to the JAK/STAT inhibitor AG490 (10 μM) ± IL-1β (10 ng/ml). Neurons were fixed and stained for neuron-specific β-tubulin III. Images are shown with **(a)** no treatment, DRG cultures treated with IL-1β for 24 h or treated with AG490 for 1 h then stimulated with IL-1 β for 24 h. In **(b)** is shown the impact on total neurite outgrowth in control and diabetic rat cultures. Data are mean ± SEM (n = 3 replicates); *p < 0.05 vs control, or AG490, or AG490+ IL-1β (two-way ANOVA with Bonferroni’s test). In (**c** and **d**) is shown total neurite outgrowth levels of DRG cultures from normal rats transfected with plasmids carrying GFP and scramble sequence (scr) or STAT3 shRNA for 48 h and then stimulated with IL-1β (10 ng/ml) for 24 h. Neurite outgrowth was derived from mean pixel area of the GFP signal captured from live cells under confocal microscopy. Data are mean ± SEM (n = 3 replicates); *p<0.05 vs scr + IL-1β, **p<0.05 vs scr + IL-1β, ***p<0.05 vs scr + IL-1β ( oneway ANOVA with Dunnett’s test). In **(e)** Western blot from DRG cultures studied in **(c,d)** that were analyzed for T-STAT3 and T-ERK protein. In **(f)** quantification of expression levels of T-STAT3/T-ERK are expressed and are means ± SEM (n = 4 replicates); *p<0.05 vs scrambled or scrambled + IL-1β ( oneway ANOVA with Dunnett’s test).

The involvement of the JAK/STAT signal transduction pathway in IL-1β -directed neurite outgrowth was tested by treating with the well characterized JAK inhibitor, AG490. In the presence of low dose of neurotrophins, cultures from normal and diabetic rats were treated with 10 μM AG490 and impact on IL-1β dependent neurite outgrowth assessed. AG490 treatment alone had little effect on neurite outgrowth in the absence of IL-1β at 24 h. In the presence of 10 ng/ml of IL-1β, there was a 3-fold elevation in neurite outgrowth that was completely blocked by AG490 (Figure 4a and b). Another approach to demonstrate the involvement of the JAK/STAT pathway was to over-express shRNA to STAT3. DRG cells were infected with lentivirus carrying scramble (scr) or shRNA to STAT3 for 48 h and stimulated with IL-1β at 10 ng/ml for 12 hrs. Figure 4e and f shows a significant but not complete decrease in STAT3 expression in the presence of shRNA to STAT3. Treatment with shRNA to STAT3 significantly, but not completely, blocked the augmentation of neurite outgrowth induced by IL-1β (Figure 4c and d).

### Endogenous IL-1β expression modulates neurite outgrowth in cultured adult sensory neurons

**Figure 5:**
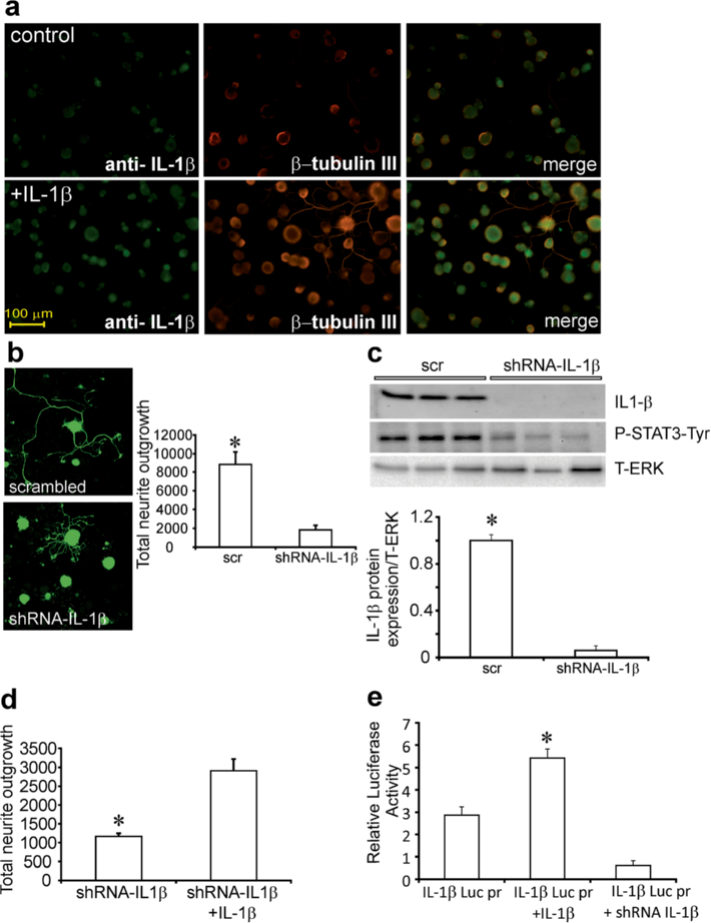
**DRG sensory neurons express IL-1β and its blockade inhibits neurite outgrowth.** In **(a)** DRG sensory neurons from normal rats were cultured for 1 day in the presence of low dose of neurotrophic growth factors and were fixed and immunostained for IL-1β. Top panels are untreated cells and bottom panels are treated with IL-1β (10 ng/ml) for 24 h. The right panels are co-stained for β-tubulin III. In **(b)** DRG cultures were infected with GFP-expressing lentivirus containing scrambled (scr) or shRNA to IL-1β. After 48 h of culture images of GFP fluorescence were acquired and neurite outgrowth quantified. Data are mean ± SEM (n = 3 replicates); *p<0.05 vs shRNA-IL-1β (Student’s *t*-Test). In addition, in **(c)** infected cultures were examined by Western blot for IL-1β expression and P-STAT3-Tyr and revealed significantly reduced expression in presence of shRNA to IL-1β. In **(d)** DRG neurons over-expressed shRNA to IL-1β for 48 h and then were treated ± IL-1β. Neurons were stimulated with/without IL-1β for 24 h and then neurite outgrowth quantified. Data are mean ± SEM (n = 3 replicates); *p < 0.05 vs shRNA for IL-1β + IL-1β (Student’s *t*-Test). In **(e)** IL-1β treatment for 24 h up-regulates IL-1β luciferase promoter activity in cultured DRG sensory neurons. Co-transfection with plasmid expressing GFP and carrying shRNA to IL-1β blocked the effect of exogenous IL-1β. Data are mean ± SEM (n = 3 replicates); *p<0.05 vs all groups (oneway ANOVA with Dunnett’s test).

We cultured adult rat DRG sensory neurons for 24 h with low dose neurotrophic factors in the presence or absence of IL-1β at 10 ng/ml. Cultures were fixed and stained using anti-IL-1β antibody and antibody to neuron-specific β-tubulin III (Figure 5a). Immunocytochemical staining for IL-1β revealed that the majority of DRG neurons expressed IL-1β in the control rat cultures. To inhibit endogenous IL-1β activity, cultured DRG neurons were transduced with lentivirus carrying GFP and either scrambled or shRNA for IL-1β (Figure 5b). GFP fluorescence signal was used to quantify axonal outgrowth, demonstrating that neurite outgrowth in neurons over-expressing shRNA to IL-1β was significantly decreased (Figure 5b). We also examined the knockdown effect of shRNA to IL-1β on IL-1β and P-STAT3-Tyr705 expression in DRG cultures by Western blot (Figure 5c). The delivery of shRNA to IL-1β was very effective in lowering endogenous IL-1β and P-STAT3-Tyr705 expression in these cultures. In DRG neurons over-expressing shRNA to IL-1β for 48 h and stimulated with IL-1β for the final 12 h there was a significant effect of exogenous IL-1β on neurite outgrowth (Figure 5d). Another approach was to use promoter reporter technology to study IL-1β transcriptional activity. IL-1β promoter reporter construct (IL-1β-Luc-pr) and/or shRNA to IL-1β were simultaneously transfected into adult sensory neuron cultures and luciferase activity assessed after 48 h. A sub-group of cultures were treated with 10 ng/ml of IL-1β for the final 12 h. Figure 5e shows that IL-1β raised IL-1β promoter activity by approximately 2-fold and shRNA to IL-1β significantly decreased this activity suggesting auto-regulation of IL-1β promoter.

### STAT3 is localized to mitochondria upon Ser727 phosphorylation triggered by IL-1β

**Figure 6:**
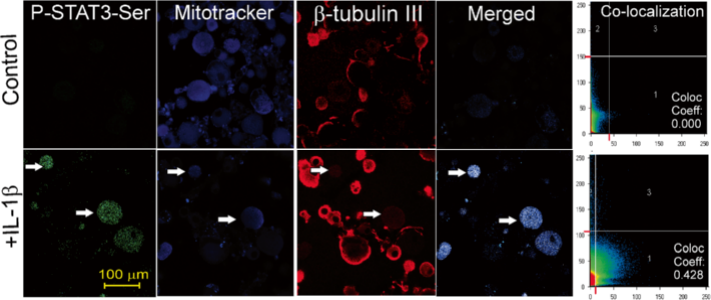
**IL-1β induces co-localization of P-STAT-Ser727 with mitochondria in cultured DRG sensory neurons.** DRG neurons were stimulated with IL-1β (10 ng/ml) for 30 min and then live cells loaded with MitoTracker deep red and then fixed and co-stained for P-STAT3-Ser727 and β-tubulin III. Confocal images of control (top panels) and IL-1β stimulated neurons (bottom panels) showing the individual channels and the co-localization coefficients between P-STAT3-Ser727 and MitoTracker deep red are shown. White arrows indicate neurons (e.g. positive for β-tubulin III) with P-STAT3-Ser727 and Mitotracker co-localization. Zeis LSM510 software was used to measure the co-localization coefficient; a threshold was set manually and a table of results was generated by the software, as described previously [68]. Co-localization analysis was based upon review of 163 ± 4.1 neurons for each treatment of which 25.3 ± 5.6 neurons (n=3 replicates) exhibited high expression of P-STAT3-Ser727. The overall coefficient for co-localization with Mitrotacker deep red for the high expressing P-STAT3-Ser727 neurons was calculated as 0.432 ± 0.086 (as derived from the Zeiss LSM510 software).

STAT3 when phosphorylated on the Ser727 epitope localized to the mitochondria of non-neuronal and clonal cells [28,30,31]. We determined if P-STAT3-Ser727 was localized to the mitochondria in response to IL-1β signaling. Adult rat DRG sensory neurons derived from normal rats were cultured for 24 h with low dose of neurotrophic factors, in the presence or absence (Figure 6) of IL-1β at 10 ng/ml. Cultures were fixed, stained for P-STAT3-Ser 727, neuron-specific β-tubulin III and Mitotracker deep red. Background levels of P-STAT3-Ser727 staining in control cultures were below detection threshold, however, a small cohort (approximately 15%) of DRG neurons stimulated with IL-1β exhibited significantly increased expression of P-STAT3-Ser727 compared to control. By confocal microscopy, we showed that IL-1β induced co-localization of P-STAT3-Ser727 and Mitotracker deep red in neurons with a co-localization coefficient of 0.43 ± 0.086.

### Bioenergetic profile is abnormal in neurons cultured from STZ-diabetic mice and was corrected by IL-1β

**Figure 7:**
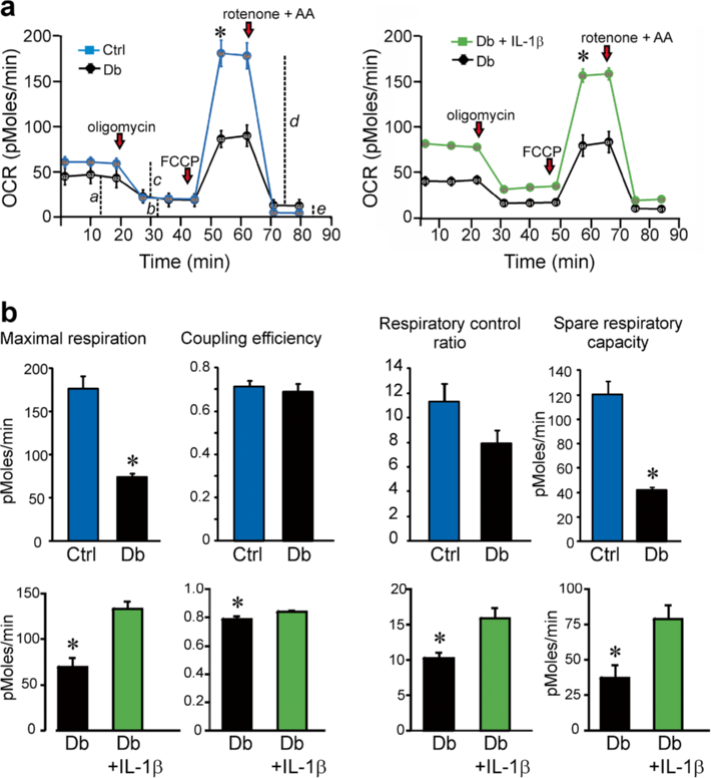
**Mitochondrial bioenergetics is abnormal in cultured neurons from diabetic mice and is corrected by IL-1β.** OCR was measured at basal level with subsequent and sequential addition of oligomycin (1 μM), FCCP (1 μM), and rotenone (1 μM) + antimycin A (AA; 1 μM) to DRG neurons cultured from age-matched control (blue) and 3–5 month STZ-induced diabetic mice (black) in the presence of low dose neurotrophic growth factors. Data are expressed as OCR in pmol/min for 1000 cells (there were approximately 2500–5000 cells per well). Dotted lines, *a–e* in **(a)** have been used later for quantification of bioenergetics parameters. The OCR measurements in **(a)** control (blue), diabetic (black), or treated with IL-1β (green) at the 1 μM concentration of FCCP were plotted. Maximal respiration (*d-e*), coupling efficiency (*c/a*), respiratory control ratio (*d/b*) and spare respiratory capacity (*d–a*) in **(b)** are presented for control (blue), diabetic (black) and diabetic treated with IL-1β (green) and were calculated after subtracting the non-mitochondrial respiration (*e*) as described [41]. Values are mean ± SEM of n = 5 replicate cultures; *p < 0.05 by Student’s t-Test.

**Figure 8:**
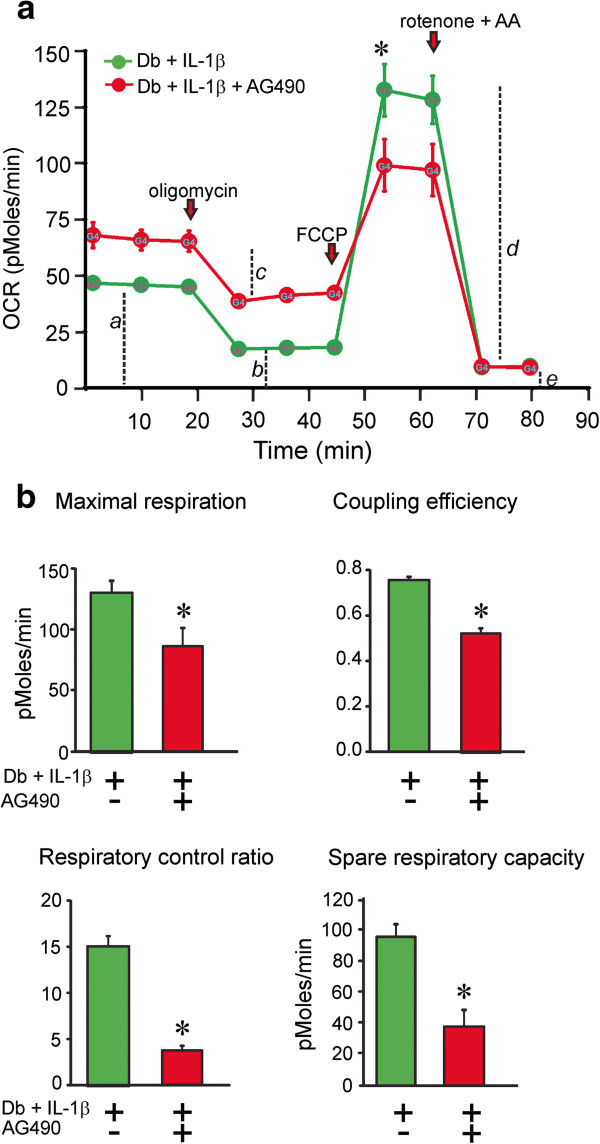
**Mitochondrial bioenergetics in cultured neurons from diabetic mice is abnormal when it is treated with AG490.** OCR was measured in the same conditions as Figure 7. DRG neurons were cultured from 3–5 month STZ-induced diabetic mice treated with IL-1β (green) or from 3–5 month STZ-induced diabetic mice treated with AG490 for 1 hr than stimulated with IL-1β (red) in the presence of low dose neurotrophic growth factors. Data are expressed as OCR in pmol/min for 1000 cells (there were approximately 2500–5000 cells per well). The OCR measurements in **(a)** diabetic treated with IL-1β (green) or diabetic treated with AG490 for 1 hr than stimulated with IL-1β (red) at the 1 μM concentration of FCCP were plotted. Bioenergetic parameters were determined as described in Figure 7 and presented in **(b)** for DB+IL-1β (green), DB+AG490+IL-1β (red). Values are mean ± SEM of n = 5 replicate cultures; *p < 0.05 by Student’s t-Test. Control cultures were treated with IL-1β for 24 hr and revealed increases in maximal respiration; control, 159.8 ± 11.7 vs IL-1β, 299.5 ± 70.0 and spare respiratory capacity; control, 116.5 ± 16.6 vs IL-1β, 191.0 ± 44.0. Values are means ± SEM, n=2-4, and expressed as pMoles/min normalized to neuron number (per 5000 cells).

To assess the mitochondrial bioenergetics profile in sensory neurons derived from DRG of 3–5 month STZ-diabetic mice, the oxygen consumption rate (OCR) was measured in neurons cultured for 24 h using the Seahorse Biosciences XF24 analyzer. In Figure 7 the OCR measurements in (a) control (blue), diabetic (black), or treated with IL-1β (green) at the 1 μM concentration of FCCP were plotted. Maximal respiration (*d-e*), coupling efficiency (*c/a*), respiratory control ratio (*d/b*) and spare respiratory capacity (*d–a*) in (b) are presented for control (blue), diabetic (black) and diabetic treated with IL-1β (green) and were calculated after subtracting the non-mitochondrial respiration (*e*) as described [41]. The coupling efficiency is determined from the change in basal respiration rate on addition of oligomycin. It is sensitive to changes in all bioenergetics modules. The respiratory control ratio is the ratio of the uncoupled rate to the rate with oligomycin and is sensitive to changes in substrate oxidation and proton leak. Spare respiratory capacity indicates how close a cell is to its bioenergetics limit when the cells are operating under stress and/or maximal metabolic flux. The maximal OCR induced by the uncoupler FCCP (1 μM) in neurons cultured from diabetic mice was significantly decreased compared to age-matched control mice and improved by IL-1β (10 ng/ml) in diabetic neurons treated for 24 h (Figure 7a). The significant impairment of maximal electron transport activity is indicative of suboptimal spare respiratory capacity in diabetic neurons compared with age matched controls. Neurons from diabetic mice cultured with IL-1β for 24 h exhibited significantly improved maximal respiration, coupling efficiency (very small impact and possibly physiologically irrelevant), respiratory control ratio and spare respiratory capacity (Figure 7b). Inhibition of the JAK/STAT pathway induced by AG490 (10 μM) for 24 h significantly suppressed maximal respiration rates, coupling efficiency, respiratory control ratio and spare respiratory capacity in IL-1β treated diabetic neurons (Figure 8a,b). In DRG cultures from normal rat treatment with IL-1β caused a small but reproducible increase in maximal respiration and spare respiratory capacity (see legend to Figure 8). This correlated with the small increases in P-STAT3 induced by IL-1β in control cultures (Figure 3d).

## Discussion

We discovered for the first time that IL-1β expression was reduced in sensory neurons and peripheral nerve in diabetes. The generalized down-regulation of cytokine expression seen both in the present study and previously [40] clearly shows that an inflammatory environment is not triggered in the DRG or nerve during early stages of experimental diabetes. Therefore, the primary aim of the present study was to investigate the signal transduction pathways utilized by IL-1β in regulating neurite outgrowth in adult sensory neurons. By understanding these events we hope to identify novel drug targets for therapy in diabetic neuropathy, a severe neurodegenerative disease involving impaired axonal plasticity. The results show that IL-1β augmented neurite outgrowth, in part, through the JAK-STAT3 pathway (the activity of this pathway was impaired in DRG isolated from diabetic animals). Blockade of JAK-STAT3 signaling using pharmacological inhibition or shRNA to STAT3 significantly reduced IL-1β dependent neurite outgrowth. We also uncovered a novel autocrine pathway whereby endogenous neuronal IL-1β enhanced neurite outgrowth. Finally, mitochondrial function was impaired in neurons derived from diabetic mice and could be up-regulated by IL-1β treatment through a JAK-STAT dependent pathway.

In the peripheral nervous system other groups have shown that IL-1β can promote neurite outgrowth from DRG neurons. In organ culture of adult DRG IL-1β enhanced axon regeneration [42]. In dissociated rat sensory neurons from postnatal day 9–10 treatment with IL-1β increased neurite outgrowth via p38 MAPK activation [6]. Delivery of IL-1β by miniosmotic pump for 2 weeks to the crushed sciatic of rats was able to accelerate axon regeneration as measured using morphological and functional criteria [15]. IL-1β knockout mice exhibit reduced functional recovery, specifically locomotor function, following nerve crush although IL-1β clearly has several complementary roles at the crush site including mediating neutrophil biology and hyperalgesia [7]. Combined with this previous literature our study reveals that treatment with the cytokines IL-1α and β enhanced neurite outgrowth via direct effects on cultured neurons. Neurons derived from normal or diabetic rats were able to respond to IL-1β with effects of IL-1β more pronounced in the absence of neurotrophic factors. This data provides encouraging evidence that in vivo treatment of damaged nerves in the setting of diabetes can be effective in enhancing axonal plasticity and ideally augmenting levels of distal nerve fibers in the skin (the primary site of fiber loss in diabetes) [43,44].

Our studies focused on IL-1β dependent signaling via the JAK-STAT3 pathway. During development of the nervous system STAT3 plays an important role in axon pathfinding, neurite outgrowth and glial cell differentiation [45]. In rodents, STAT3 expression is detected in neurons and glia from embryonic day 14 to postnatal in rat brain [46]. STAT3 is an interesting transcription factor in the context of axon regeneration in the peripheral nervous system. STAT3 expression and phosphorylation on Tyr-705 was enhanced in neurons and proximal nerve following crush injury [22,23]. Intriguingly, STAT3 activation, in part, through the JAK2 signaling pathway occurs in the axons and perikarya of DRG neurons after peripheral, but not central lesion, strongly supporting a role for STAT3 in sensory axon regeneration [18,19,23]. cAMP through induction of neuropoietic molecules such as IL-6, LIF and CNTF activates STAT3 (phosphorylated on Tyr-705) as a component of a conditioning injury in the DRG [47]. The appearance of P-STAT3-Tyr705 in the perikarya is a dual leucine zipper kinase (DLK) and JIP3 dependent process involving retrograde transport of activated STAT3 from the site of injury [48]. Activated STAT3 within the perikarya initiates a range of transcriptional changes that drives peripheral axon regeneration (reviewed by [2,49]). In addition, activation of STAT3 within the axon can afford neuroprotection from axotomy-induced axonal degeneration [50]. Our work reveals that STAT3 can be activated rapidly by IL-1β in vitro and the process of activation involves phosphorylation on Tyr-705. Furthermore, for the first time in adult primary neurons we show that phosphorylation on Ser-727 occurs (which could be linked to IL-1β mediated optimization of mitochondrial bioenergetics – see later).

Sensory neurons of the DRG express IL-1β although ability to secrete the protein remains unclear [12]. We show that cultured sensory neurons expressed IL-1β and exhibited endogenous transcriptional activity for the IL-1β promoter (Figure 5). Blockade of endogenous IL-1β reduced neurite outgrowth thus revealing for the first time an autocrine pathway for local control of axonal plasticity by endogenous IL-1β in adult neurons. Table 1 shows that endogenous IL-1β expression was reduced in DRG and nerve of diabetic animals. In CNS neurons, IL-1β enhances expression and secretion of IL-6 and GDNF [51-54] so that down-regulation of IL-1β expression in diabetes could trigger a generalized sup-optimal neurotrophic environment. The down-regulation of cytokine gene expression and synthesis is an early target of diabetes that is likely to have pathogenic consequences [55].

The diabetes-induced impairments in IL-1β signaling were linked to aberrant mitochondrial bioenergetics. We used Seahorse Biosciences XF24 analysis to measure cell respiratory control, recording rate of ATP production, proton leak, coupling efficiency, maximum respiratory rate, respiratory control ratio and spare respiratory capacity [41]. Measurements of oxygen consumption rate in the presence of uncoupler revealed that the maximal electron transport capacity was significantly depressed in sensory neurons from STZ-diabetic mice. In our study, phosphorylation of the STAT3 on Ser-727 and the neuronal mitochondrial bioenergetics profile were impaired in DRG of STZ-diabetic mice and rats and this was prevented by IL-1β treatment. We believe these defects in mitochondrial function detected by our bioenergetics analysis were not due to a general loss of mitochondrial number or mass in the cultured cells since our previous work reveals no loss of mitochondrial mass [38,39,56]. This data complements our recent findings that diabetes induces mitochondrial abnormalities and dysfunction in sensory neurons in type 1 diabetes [33,38,39,56] and reviewed [33]. Data in Figures 3 and 6 show that STAT3 phosphorylation on Ser-727 was impaired by diabetes and enhanced by IL-1β treatment. In Figure 6 we show localization of P-STAT3-Ser727 to the mitochondria. Previous work shows that STAT3 is involved in cellular respiration by regulating the activity of mitochondrial complexes I and II of the electron transport system when phosphorylated on Ser727 [28,30,31]. STAT3 was observed to localize in mitochondria and interact with complex I components and GRIM 19 [57,58]. The mitochondrial activity of STAT3 was confined to serine phosphorylation at position 727, as overexpression of mutant STAT3-S727A in STAT3 −/− cells did not restore the mitochondrial complex I activity [28]. Thus, activated STAT3 targets multiple sites within the axon, including the microtubule network [50] and mitochondria [31], to optimize function and enhance axonal plasticity subsequent to damage.

## Conclusions

This study demonstrates a novel role for of IL-1β in preventing the diabetic phenotype of sensory neurons. This work confirms previous studies demonstrating diabetes-induced reductions in peripheral nerve tissue of cytokines such as IL-6, TNFα and CNTF [40,59,60]. The mechanism of action of IL-1β was mediated, in part, via optimization of mitochondrial bioenergetics through the JAK/STAT pathway, possibly encompassing a novel pathway linked to STAT3 modulation of mitochondrial function. We propose that a generalized down-regulation of cytokine expression leading to sub-optimal axonal plasticity at distal nerve sites contributes to development of sensory neuropathy in early stages of experimental diabetes.

## Materials and methods

### Induction of type 1 diabetes in rodents

Male Sprague Dawley rats were made diabetic with a single intraperitoneal injection of 75 mg/kg streptozotocin (STZ; Sigma, St Louis, MO, USA) and male outbred Swiss Webster mice were made diabetic by injection of 90 mg/kg STZ on two consecutive days, with each injection preceded by a 12 h fast. Only animals with blood glucose levels of > 19 mM at the start and end of the study were retained as diabetic. Sensory neuropathy was confirmed in rodents at 2 months using loss of thermal sensitivity as a marker (data not shown). Tissue was collected from rodents after 2–5 months of diabetes. Animal procedures followed guidelines of University of Manitoba Animal Care Committee using Canadian Council of Animal Care rules.

### Sensory neuron cultures and treatments

DRG from adult male rats or mice were dissociated using previously described methods [61-63]. Neurons were cultured in defined Hams F12 media in the presence of modified Bottensteins N2 supplement without insulin (0.1 mg/ml transferrin, 20 nM progesterone, 100 mM putrescine, 30 nM sodium selenite 1.0 mg/ml BSA; all additives were from Sigma, St Louis, MO, USA; culture medium was from Life Technologies, Grand Island, NY, USA). In some experiments the media was also supplemented with a low dose cocktail of neurotrophic factors (0.1 ng/ml NGF, 1.0 ng/ml GDNF, 1.0 ng/ml NT-3, and 0.1 nM insulin – all from Promega, Madison, WI, USA). This treatment improved viability of cultures and attempted to mimic the levels of neurotrophic support experienced in vivo by sensory neurons. Normal neurons were cultured in the presence of 10 mM glucose and 0.1 nM insulin and diabetic neurons with 25 mM glucose and zero insulin. Recombinant human IL-1β and recombinant human IL-1α were obtained from Peprotech (Cedarlane, USA). Rabbit anti-rat IL-1β antibody was from Millipore (CA, USA). AG490 was purchased from Millipore (Canada).

### Measurement of cytokine protein levels using Luminex system

Levels of cytokines (IL-1α, IL-1β, IL-10 and IL-2) were measured using a Bio-Plex Rat 9-Plex kit (Bio-Rad, Hercules, CA, USA) according to manufacturer’s instructions. Studies on rats were performed to ensure enough protein for detection. Lumbar DRG, mid sciatic nerve and tibial nerve tissue samples from control and STZ-induced diabetic rats were homogenized on ice with a polytron in the presence of Tissue Extraction Reagent I (Invitrogen, #FNN0071A). Multiplex beads were added to each well of a 96-well plate and 100 μl of either the standards or homogenized samples were added, agitated for 30 min then washed 3 times. Beads were incubated with the detection antibodies for 30 min and data were quantified on a Bio-Rad Bioplex 200 Luminex system and analyzed with associated software (Bio-Rad, Hercules, CA, USA). Samples were assayed in triplicate, while cytokine standards were in duplicate. A minimum of 100 beads per analyte was used and the levels (pg/mg) of the cytokines were calculated from the standard curve and corrected to the protein concentration.

### shRNA knockdown of gene expression in DRG neurons

For shRNA-based gene silencing, GFP expressing clones specific for IL-1β and STAT3 (clone Id: V3LMM_471275 and V3LMM_424801, respectively) were obtained from OpenBiosystems (Lafayatte, CO, USA). The pGIPz lentiviral system from the OpenBiosystems database is held at University of Manitoba, Winnipeg, Canada. A control scrambled shRNA unrelated to IL-1β and STAT3 sequence was used as a negative control for lentiviral transduction and plasmid transfection [64]. For gene silencing studies that were focused on determination of gene expression rat DRG cells were transduced with lentivirus at 20X infectious unit in the presence of polybrene (8 μg/ml) for 2 h at 37°C, complete medium was added and neurons cultured for additional 48 h. For neurite outgrowth studies neurons were transduced with lentivirus or transfected with plasmid (as described in next section). Fluorescence images derived from GFP-expressing virally transduced or plasmid transfected neurons were acquired by using a LSM510 confocal microscope (Carl Zeiss) with 20X air objective.

### Luciferase reporter constructs for IL-1β and cell transfection

Reporter plasmid with the IL-1β promoter upstream from luciferase was kindly donated by Dr. Jian Fei (Shanghai Research Center for Model Organisms, Shanghai, China) [65]. Rat DRG cells (30×10^3^) were transfected in triplicate with 1.8 μg of IL-1β Luc-promoter plasmid DNA and 0.2 μg of pCMV-Renilla (Promega, Madison, WI, USA) using the Amaxa Nucleofector electroporation kit for low numbers of cells according to the manufacturer’s instructions (ESBE Scientific, Toronto, ON, Canada). In some experiments co-transfection was performed with plasmid carrying shRNA to STAT3 (clone ID: V3LMM_424801) or shRNA to IL-1β (clone ID: V3LMM_471275) obtained from OpenBiosystems (Lafayatte, CO, USA). Cells were lysed using passive lysis buffer provided with the Dual-Luciferase Reporter Assay System (Promega, Madison, WI, USA). The luciferase activity was measured using a luminometer (model LMAXII; Molecular Devices, Sunnyvale, CA, USA). 20 μl of each sample was loaded in a 96-well plate and was mixed with 100 μl of Luciferase Assay Reagent II and firefly luciferase activity was first recorded. Then, 100 μl of Stop-and-Glo Reagent was added, and Renilla luciferase activity was measured. All values are normalized to Renilla luciferase activity. For lentiviral infection or plasmid transfection cultures of rat cells were preferred due to high cell yields.

### Quantification of neurite outgrowth

GFP-based or immunostaining-based fluorescent images were captured and the mean pixel area determined using ImageJ software (adjusted for the cell body signal). All values were normalized for neuronal number. In this culture system the level of total neurite outgrowth has been previously validated to be directly related to an arborizing form of axonal plasticity and homologous with in vivo collateral sprouting [66]. Studies were performed with rat cultures due to higher cell yields.

### Western blotting

Cultured DRG neurons from adult rats were harvested after 24 h or intact lumbar DRG from adult mice were rapidly isolated and then homogenized in ice-cold stabilization buffer containing: 0.1 M Pipes, 5 mM MgCl2, 5 mM EGTA, 0.5% Triton X-100, 20% glycerol, 10 mM NaF, 1 mM PMSF, and protease inhibitor cocktail [67]. Proteins were assayed using DC protein assay (BioRad; Hercules, CA, USA) and Western blot analysis performed as previously described [39,67]. The samples (5 μg total protein/lane) were resolved on a 10% SDS-PAGE gel, and electroblotted (100 V, 1 h) onto a nitrocellulose membrane. Blots were then blocked in 5% nonfat milk containing 0.05% Tween overnight at 4°C, rinsed in TBS-T and then incubated with the primary antibodies for phospho-STAT3 (Tyr705) or phospho-STAT3 (Ser727) or T-STAT3 antibody (1:1000; Cell Signaling Technology, Danvers, MA, USA) or IL-1RAcP (1:500; Santa Cruz Biotechnologies, CA). Total ERK (1: 1500; Santa Cruz Biotechnologies, CA) was used as a loading control. After four washes of 10 min in TBS-T, secondary antibody was applied for 1 h at room temperature. The blots were rinsed, incubated in Western Blotting Luminol Reagent (Santa Cruz Biotechnologies, CA, USA), and imaged using the BioRad Fluor-S Max image analyzer. For culture work source material was from rats to ensure high enough protein yields. DRG samples were collected from control vs diabetic mice since the Swiss Webster mice were cheaper to maintain, reveal less inter-animal variability and onset of neuropathy was quicker compared with rats.

### Co-localization studies and immunocytochemistry for detection of tubulin and phospho-STAT3 (Tyr705 or Ser727)

For co-localization experiments DRG neuron cultures from adult rats were incubated with 500 nM Mitotracker deep red TM (Molecular Probes, Invitrogen, USA) at 37°C for 30 min. Cultures were fixed with 4% paraformaldehyde in phosphate buffered saline (PBS, pH 7.4) for 15 min at room temperature then permeabilized with 0.3% Triton X-100 in PBS for 5 min. Cells were then incubated in blocking buffer (Roche, Indianapolis, IN, USA) diluted with FBS and 1.0 mM PBS (1:1:3) for 1 h then rinsed three times with PBS. Primary antibodies used were: β-tubulin isotype III (1:1000) neuron specific from Sigma Aldrich, Oakville, ON, Canada; phospho-STAT3 (Tyr705 or Ser727 (1:300) Cell Signaling Technology, Danvers, MA, USA). Antibodies were added to all wells and plates were incubated at 4°C overnight. The following day, the coverslips were incubated with FITC- and CY3-conjugated secondary antibodies (Jackson ImmunoResearch Laboratories, West Grove, PA, USA) for 1 h at room temperature, mounted and imaged using a Carl Zeiss Axioscope-2 fluorescence microscope equipped with an AxioCam camera. Images were captured using AxioVision3 software. Co-localization coefficients between P-STAT3-Ser727 and MitoTracker deep red were analyzed using confocal imaging with Zeiss LSM510 software and followed procedures previously described [68].

### Measurement of mitochondrial respiration in cultured DRG neurons from mice

An XF24 Analyzer (Seahorse Biosciences, Billerica, MA, USA) was used to measure neuronal bioenergetic function. The XF24 creates a transient 7 μl chamber in specialized 24-well microplates that allows for oxygen consumption rate (OCR) to be monitored in real time. Culture medium was changed 1 h before the assay to unbuffered DMEM (Dulbecco’s modified Eagle’s medium, pH 7.4) supplemented with 1 mM pyruvate, and 10 mM D-glucose. Neuron density in the range of 2,500-5,000 cells per well gave linear OCR. Oligomycin (1 μM), carbonylcyanide-p-trifluoromethoxyphenylhydrazone (FCCP; 1.0 μM) and rotenone (1 μM) + antimycin A (1 μM) were injected sequentially through ports in the Seahorse Flux Pak cartridges. Each loop was started with mixing for 3 min, then delayed for 2 min and OCR measured for 3 min. This allowed determination of the basal level of oxygen consumption, the amount of oxygen consumption linked to ATP production, the level of non-ATP-linked oxygen consumption (proton leak), the maximal respiration capacity and the non-mitochondrial oxygen consumption [41,69]. Oligomycin inhibits the ATP synthase leading to a build-up of the proton gradient that inhibits electron flux and reveals the state of coupling efficiency. Uncoupling of the respiratory chain by FCCP injection reveals the maximal capacity to reduce oxygen. Finally, rotenone + antimycin A were injected to inhibit the flux of electrons through complexes I and III, and thus no oxygen was further consumed at cytochrome c oxidase. The remaining OCR determined after this intervention is primarily non-mitochondrial. Following OCR measurement the cells were immediately fixed and stained for β-tubulin III as described above. The plates were then inserted into a Cellomics Arrayscan-VTI HCS Reader (Thermo Scientific, Pittsburgh, PA, USA) equipped with Cellomics Arrayscan-VTI software to determine total neuronal number in each well. Data are expressed as OCR in pmoles/min for 1,000 cells. Cultures from control vs diabetic mice were used since the Swiss Webster mice were cheaper to maintain, reveal less inter-animal variability and onset of neuropathy was quicker compared with rats.

### Statistical analysis

Where appropriate, data (presented as mean ± SEM) were subjected to one-way ANOVA with post-hoc comparison using Dunnett’s t test (for dose response studies) or Tukey’s tests or regression analysis with a one-phase exponential decay parametric test with Fisher’s parameter (GraphPad Prism 4, GraphPad Software Inc., San Diego, CA). Where appropriate two-way ANOVA was performed with Bonferronis post hoc test. In all other cases two-tailed Student’s t-Tests were performed.

## Abbreviations

IL-1β: Interleukin-1 beta; DRG: Dorsal root ganglia; STZ: Streptozotocin; NGF: Nerve growth factor; scr: Scramble; OCR: Oxygen consumption rate.

## Competing interests

The authors declared that they have no competing interests.

## Authors’ contributions

A.S. performed research, designed experiments, analyzed data and wrote the paper. S.R.C., D.S., S.B., L.T., A.B., R.V.P. performed experiments. E.S. analyzed data. P.F. designed experiments, analyzed data and wrote the paper. All authors read and approved the final manuscript.
